# A Proof-of-Concept Electrochemical Cytosensor Based on *Chlamydomonas reinhardtii* Functionalized Carbon Black Screen-Printed Electrodes: Detection of *Escherichia coli* in Wastewater as a Case Study

**DOI:** 10.3390/bios12060401

**Published:** 2022-06-10

**Authors:** Amina Antonacci, Fabiana Arduini, Raouia Attaallah, Aziz Amine, Maria Teresa Giardi, Viviana Scognamiglio

**Affiliations:** 1 Institute of Crystallography, National Research Council, Department of Chemical Sciences and Materials Technologies, Via Salaria km 29.300, 00015 Monterotondo, Italy; amina.antonacci@ic.cnr.it (A.A.); fabiana.arduini@uniroma2.it (F.A.); mariateresa.giardi@ic.cnr.it (M.T.G.); 2 Department of Chemical Science and Technologies, University of Rome “Tor Vergata”, Via della Ricerca Scientifica, 00133 Rome, Italy; 3 SENSE4MED, via Renato Rascel 30, 00128 Rome, Italy; 4 Faculty of Sciences and Techniques, Hassan II University of Casablanca, Casablanca 20000, Morocco; raouia.attaallah@gmail.com (R.A.); azizamine@yahoo.fr (A.A.); 5 Biosensors S.r.l., Via degli Olmetti 44, Formello, 00060 Rome, Italy

**Keywords:** algal cytosensor, pathogen detection, wastewater, carbon black, screen-printed electrodes

## Abstract

Herein, we report a proof-of-concept algal cytosensor for the electrochemical quantification of bacteria in wastewater, exploiting the green photosynthetic alga *Chlamydomonas reinhardtii* immobilized on carbon black (CB) nanomodified screen-printed electrodes. The CB nanoparticles are used as nanomodifiers, as they are able to sense the oxygen produced by the algae and thus the current increases when algae are exposed to increasing concentrations of bacteria. The sensor was tested on both standard solutions and real wastewater samples for the detection *Escherichia coli* in a linear range of response from 100 to 2000 CFU/100 mL, showing a limit of detection of 92 CFU/100 mL, in agreement with the maximum E. coli concentration established by the Italian law for wastewater (less than 5000 CFU/100 mL). This bacterium was exploited as a case study target of the algal cytosensor to demonstrate its ability as an early warning analytical system to signal heavy loads of pathogens in waters leaving the wastewater treatment plants. Indeed, the cytosensor is not selective towards *E. coli* but it is capable of sensing all the bacteria that induce the algae oxygen evolution by exploiting the effect of their interaction. Other known toxicants, commonly present in wastewater, were also analyzed to test the cytosensor selectivity, with any significant effect, apart from atrazine, which is a specific target of the D1 protein of the Chlamydomonas photosystem II. However, the latter can also be detected by chlorophyll fluorescence simultaneously to the amperometric measurements. The matrix effect was evaluated, and the recovery values were calculated as 105 ± 8, 83 ± 7, and 88 ± 7% for 1000 CFU/100 mL of *E. coli* in Lignano, San Giorgio, and Pescara wastewater samples, respectively.

## 1. Introduction

Currently, water resources face severe quantitative and qualitative threats due to the industrialization and rapid economic development in many areas worldwide. Issues related to the ecological, health, and hygienic state of the water bodies are well-documented and represent a crucial concern to minimize the human impact on both fresh and saltwater bodies [1]. One crucial point in preserving the quality of bodies of water is wastewater management. Many quality indicators are routinely used to measure the different characteristics of disposing or reusing waste fluids. The most prominent parameters to monitor are reported in the Directive 2006/118/EC of the European Parliament on the protection of groundwater against pollution and deterioration [2].

Commonly approved techniques to monitor wastewater contaminants (e.g., pesticides, herbicides and fertilizers among others) are laboratory-based ones, which require frequent calibration, sample pre-treatment, and long and expensive procedures. Furthermore, sampling happens only at fixed intervals of time, meaning that high pollution phenomena occur in a short window of time (e.g., heavy rain that washes away pesticides/herbicides/fertilizers from cultivated fields or an accidental or malicious release of toxic compounds from an industry) may go undetected. These aspects support the necessity for novel analytical tools with similar or better sensitivity when compared to currently approved techniques but, which are at the same time, portable and easy to integrate directly on-site, with on-line detection of multi-pollutants with minimal or no pretreatment.

Biosensors offer numerous advantages compared to classical monitoring techniques. Being smaller and cheaper, they require less maintenance and show a lower frequency of calibration. This makes biosensors easily integrated into water and wastewater treatment plants, before and after the treatment process, allowing for precise and fast estimation of the incoming pollutant’s load, the efficiency of the applied treatments, and the pollutant’s load in the effluent waters. In turn, these properties allow for real-time corrections to the treatment process or, in case of heavy loads of pollutants or the presence of species difficult to degrade, a shutdown of the plant outstream to prevent downstream pollution. Due to the flexibility of both the transduction method and sensing material, biosensors can be easily adapted to detect a wide range of water parameters and pollutant species presence and concentration [3,4,5,6,7,8,9,10]. A large number of algal biosensors have also been described in the literature [11], based on diverse transduction systems [12], support materials [13], and nanomaterials [14], towards many target analytes, including herbicides [15], endocrine-disrupting chemicals [16], and chemical warfare agents [17]. A recent requirement that interrogates the algal biosensor is the detection of pathogens in wastewater, where the concentration of bacteria can be compatible with the algae sensitivity. Indeed, diverse matrices must comply with the limits imposed by European regulations for the presence of pathogens. As an example, the EU requires that “foodstuffs must not contain residue levels of veterinary medicines or biocidal products that might represent a hazard to the health of the consumer” [18]. Similar regulations have been promulgated for surface waters (e.g., lakes, rivers, and seawater) [2,19,20] and concerning wastewater treatment, with particular attention to the contaminant levels in the water leaving the treatment plant [21].

Current biosensing methods for the detection of microorganisms in various matrices are mostly centered on the antigen–antibody interaction, which in turn triggers variation in the physic-chemical parameters recorded by the transducer. These methods are very effective for monitoring microorganisms in fresh and saltwater but suffer when applied in wastewater due to the complexity, both chemical and physical, of the matrix involved. On the other hand, bacteria present in wastewater could be monitored by leveraging the effect of their interaction with certain microalgae species, e.g., algae. The beneficial effects of bacteria–algae co-cultures have been recently demonstrated, with bacteria being considered as an important source of vitamins, organic growth factors, and CO_2_, crucial for algal development, especially during carbon limitation periods [22]. However, the amount of CO_2_ produced by bacteria would be lower than the algal demand [23]. Further studies highlighted that “aerobic bacteria can promote algal growth by reducing the photosynthetic oxygen tension within the microenvironment of the algal cells” [24], boosting in turn algae to augment oxygen evolution.

Based on these findings, we hypothesized that by integrating algae cells of *Chlamydomonas reinhardtii,* immobilized on screen-printed electrodes nanomodified with carbon black (CB), in an electrochemical transduction system, we would be able to record the rising currents under light illumination and the applied potential, due to algae oxygen production in the presence of bacteria. Indeed, we demonstrated in our previous article [14] the capability of CB nanoparticles to “sensitively sense changes in algae oxygen evolution during the photosynthetic process”. To the best of our knowledge, this is the first cytosensor developed for pathogen detection that uses algae as bioreceptors.

## 2. Materials and Methods

### 2.1. Chemicals

All reagents were purchased as high purity grade. Tris-acetate-phosphate, tricine, sucrose, methanol, sodium alginate, sodium chloride, calcium chloride, atrazine, and catechol, bisphenol A were purchased from Sigma-Aldrich (St. Louis, MO, USA). Copper (Cu^2+^) and arsenic (AsIII) were purchased from Carlo Erba. Carbon black nanomodified screen-printed electrodes (CB-SPEs) were delivered by SENSE4MED, Italy.

### 2.2. Algae Growth Conditions and Physiological Characterization of the Algal Liquid Cultures

Algae growth and characterization was accomplished according to the protocols described in Ref. [14]. *C. reinhardtii* F255N was grown under continuous light (50 µL photons m^−2^ s^−1^), on tris-acetate-phosphate (TAP) culture medium into an orbital shaker at 25 °C, stirring at 150 rpm. At 72 h inoculation, the algae culture was diluted in TAP to an optical density of 0.15 OD_750_. Then the refreshed culture was grown under the same conditions for all the periods of the physiological characterization, using cell culture in an early mid-exponential growth phase, with Abs_750_ 0.5 O.D., 10^6^ cells/mL, and 5 µg/mL chlorophyll content. Cell number was quantified using a Bio-Rad TC-10 automated counter (Hemel Hempstead, UK), using a 10 µL-volume cell counting slide. Pigment content was spectrophotometrically measured by quantifying the absorbance (O.D.) of the chlorophylls a and b at 652 nm wavelength, once extracted with 80% acetone. The calculation of the total chlorophyll concentration expressed as µg/mL was performed by the equation (O.D._652_ × 1000)/34.5. The photosynthetic profile was assessed by the chlorophyll a fluorescence induction (Kautsky) curves, recorded with a Plant Efficiency Analyzer (PEA) at room temperature after 10 min of dark adaptation and with a 5 s saturating pulse excitation light (3500 µL photons m^−2^ s^−1^) using an array of six red light-emitting diodes (650 nm peak). Kautsky curves or OJIP curves are defined by a polyphasic fluorescence rise in time, with O as the minimal dark-acclimated fluorescence level (indicating that all QA are oxidised) and P as the maximal level (indicating that all PSII quinone acceptors are fully reduced). The difference in the fluorescence signal of these distinct states helps to evaluate the PSII functionality through the following parameters calculated by the fluorimeter:

✓ F_0_ or fluorescence in the initial state: minimum fluorescence intensity in the state acclimated to the darkness, recorded when all PSII reaction centres are open (oxidized quinones);
✓ F_M_ or maximum fluorescence: maximum fluorescence intensity reached after 10 min of darkness and a subsequent saturating light pulse, recorded when all reaction centres of the PSII are closed (reduced);
✓ F_V_/F_M_: maximum fluorescence yield of PSII photochemical reaction expressed as a ratio of variable fluorescence (F_M_ − F_0_) and maximum fluorescence, calculated according to the Equation (1):

F_V_/F_M_ = (F_M_ − F_0_)/F_M_, (1)

where F_V_ represents the maximum variable fluorescence calculated as F_M_-F_0_, F_M_ corresponds to the maximum fluorescence emission and F_0_ is the minimum fluorescence emission. It reflects the efficiency of PSII in using light for photochemical conversion and its value is usually at 0.8 in physiological conditions or decreased values under stress.

Optical microscopy was conducted on liquid cultures of *C. reinhardtii* algae cells in TAP medium at different grow stages, using a Leitz Diavert Microscope.

### 2.3. Algae Immobilization Protocol

Algae immobilization was accomplished according to the protocols described in Ref. [14] with a final cell concentration of 0.08 × 10^6^ cells/µL for each SPE. *C. reinhardtii* F255N cell cultures in an early mid-exponential growth phase, with Abs_750_ 0.7 O.D., 10^7^ cells/mL, and 10 µg/mL chlorophyll content, were exploited for the immobilization on carbon black modified screen-printed electrodes (CB-SPEs) purchased from SENS4MED (Rome, Italy). Algae/CB-SPEs were stored in 50 mM Tricine, 20 mM CaCl_2_, 5 mM MgCl_2_, 50 mM NaCl, and 70 mM sucrose pH 7.2 and incubated for 2 h under continuous light (50 µL photons m^−2^ s^−1^) and 25 °C. SEM analysis was conducted on a ZEISS EVO MA10 scanning electron microscope. Algae were subjected to gold metallization and then dehydration under vacuum before SEM analysis. Microphotographs were provided at a magnification of 2 µm.

### 2.4. Biosensor Prototype

**Figure 1:**
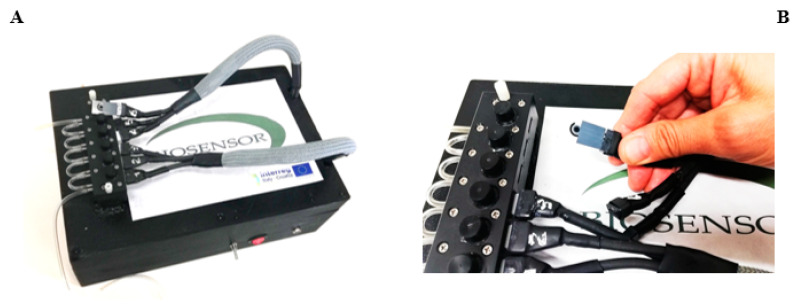
(**A**) Algal cytosensor prototype equipped with six measurement cells, fluidics, and the opto-electrochemical set-up, (**B**) Screen-printed electrode ready for the insertion into the measurement cell. Dimensions of the instrument: L28.5 × I19 × H10 cm.

A dual electro-optical transduction prototype was projected and realized to furnish both optical and electrochemical analysis by the company Biosensor Srl (Figure 1A). The instrument is a portable prototype consisting of 6 module chambers for the insertion of the algal CB-SPEs (Figure 1B). The chamber is equipped with a LED system (of 350 μL photons m^−2^ s^−1^ of red light at a 650 nm wavelength) that provides the algae illumination. The electrochemical set-up is constituted of a DC voltage supply, which provides a bias potential in the range of ±0.800 V between the working and the reference electrodes, and an amperometer to detect the current intensity variation deriving from the algae oxygen evolution process. The biological module, perfectly sealed, hosts the samples under test. Both static and dynamic operations are allowed thanks to an automatically controlled fluidic system equipped with inlet/outlet connections for the electrolytic/washing solution and sample flow. Fifty mM Tricine, 20 mM CaCl_2_, 5 mM MgCl_2_, 50 mM NaCl, 70 mM sucrose, and pH 7.2 was used as the measuring buffer for the electrochemical analysis.

### 2.5. Pathogen Detection

The electrochemical detection of *Escherichia coli* (*E. coli* BL21), exploited as a case study pathogen, was provided by following algae oxygen evolution capacity at an applied potential of −0.6 V, using a dual electro-optical transducer prototype (Biosensor Srl, Via degli Olmetti, Rome, Italy). Algae were illuminated by a 350 µL photons m^−2^ s^−1^ light with repeated cycles of 30 s light excitation and 10 min dark. An applied potential of −0.6 V was used with an acquirement interval of 0.5 s. Pathogens were added into the electrochemical chamber (200 µL volume containing 50 mM Tricine, 20 mM CaCl_2_, 5 mM MgCl_2_, 50 mM NaCl, 70 mM sucrose pH 7.2) in a concentration range from 100 to 2000 CFU/100 mL and the current signals, due to oxygen production on the CB-SPE working electrode, were recorded in dependence to the target analyte concentrations.

## 3. Results

### 3.1. Effect of Wastewater Samples on the Alga C. Reinhardtii

With the aim to design an algal cytosensor for pathogen detection, the first step entailed the study of the effect of wastewater samples on the algal physiological parameters including the photosynthetic activity, the growth rate, and the pigment content. The green photosynthetic alga C. reinhardtii was thus grown in different water samples from 3 selected sites in the Adriatic region, i.e., Lignano, San Giorgio, and Pescara depuration plants (DPs).

**Figure 2:**
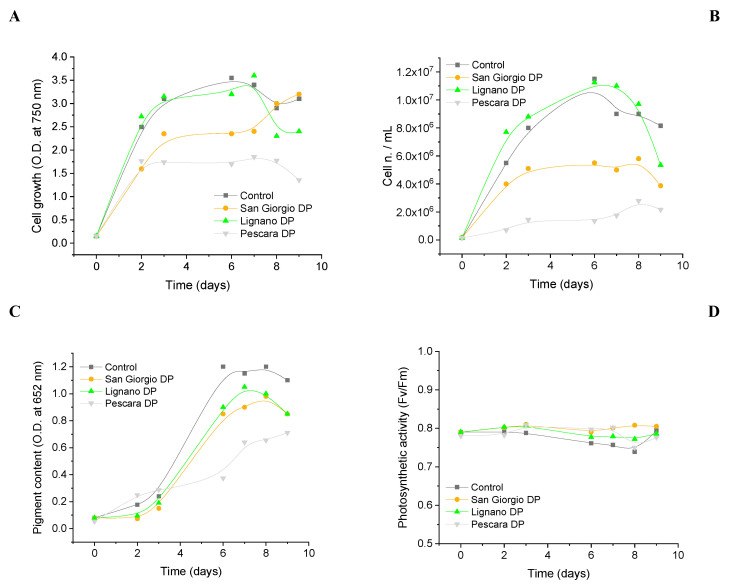
*C. reinhardtii* physiological characterization. Cell culture growth reported as absorbance at 750 nm (**A**) and cell number/mL (**B**). (**C**) Chlorophyll content. (**D**) Maximum fluorescence yield FV/FM calculated on each Kautsky curve. Incubation time: 9 days under continuous light (50 µL photons m^−2^ s^−1^). Average values ± SE (*n* = 3).

In detail, optical density, cell number, chlorophyll a fluorescence, and the total chlorophyll content were measured. Results on growth (absorption at 750 nm and cell number/mL, Figure 2A,B) and pigment content (Figure 2C) evidenced a slight influence of the Lignano water sample on algae cell grow in terms of altered vital processes and variations in the physiological parameters (e.g., cell duplication). On the contrary, a toxic effect from San Giorgio and Pescara water samples was evidenced on both algae growth (Figure 2A,B) and pigment content production (i.e., chlorophylls) (Figure 2C). The photochemical efficiency of PSII was also evaluated through Kautsky curves as described in Section 2.3 “Algae physiological characterization”, following the maximum fluorescence yield of Photosystem II F_V_/F_M_ during the analyzed period of 9 days (Figure 2D). In this case, no effect was registered regarding the maximum fluorescence yield, which remains constant during the time.

**Figure 3:**
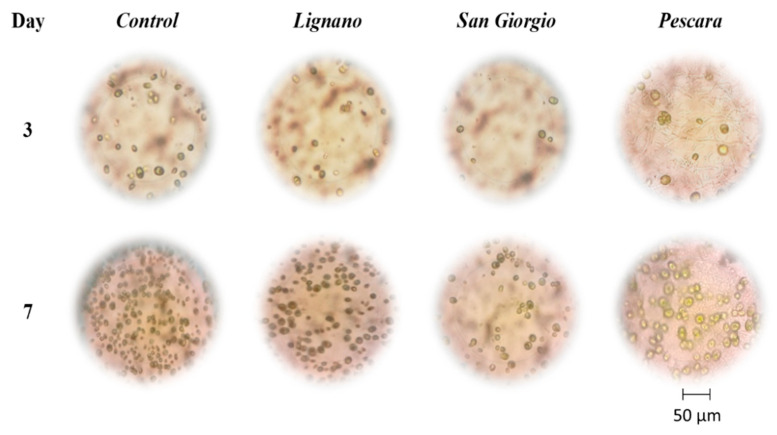
Optical microscopy images of algae cells grown in control medium and wastewater samples from depuration plants in Lignano, San Giorgio, and Pescara (Italy).

Optical microscopy images also evidenced a slowdown of the cell growth in the time for algae incubated in San Giorgio and Pescara water samples, in comparison with the control, while any effect was observed in the presence of Lignano water (Figure 3).

### 3.2. Set-Up of the Algal Cytosensor and Assessment of the Analytical Parameters

**Figure 4:**
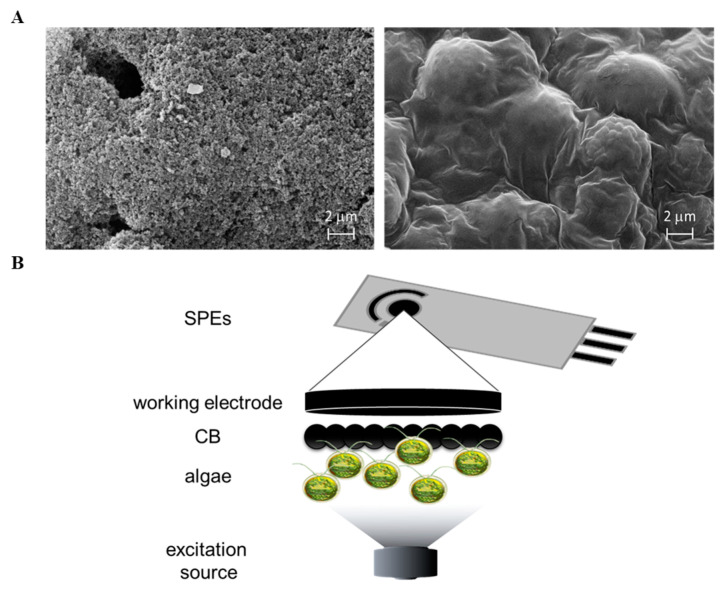
(**A**) SEM microphotographs of CB modified SPEs (left) and CB modified SPEs functionalized with algae whole cells entrapped into the calcium/alginate matrix (right). (**B**) Scheme of the obtained cytosensor for pathogen detection in wastewater. (**B**) Scheme of the proposed algal/CB-SPE cytosensor.

The algal CB-SPEs and CB-SPEs immobilized with algae (using the protocol described in Section 2.3) were observed at Scanning Electron Microscopy (SEM) as reported in Figure 4A, which show microphotographs of CB (left) and algae whole cells entrapped into the calcium/alginate matrix (right) at a magnification of 2 μm. The algal/CB-SPEs were thus inserted into the measurement chamber of the biosensor prototype (Figure 1) and amperometric measurements were accomplished for the detection of E. coli, a case study pathogen that can be found in wastewater. A scheme of the obtained algal/CB-SPE cytosensor was reported in Figure 4B.

**Figure 5:**
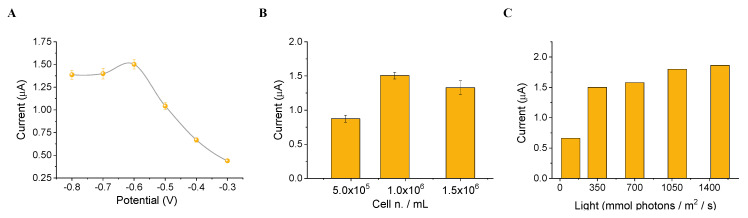
(**A**) Current intensity vs. applied potential. (**B**) Current intensity vs. immobilized cell number. (**C**) Current intensity vs. light intensity.

Once we have obtained the algal/CB-SPE cytosensor, all the analytical parameters were optimized. The best applied potential was set for monitoring the oxygen reduction signal generated by the algal activity both in the absence and in the presence of the target, in response to light exposure (λ = 650 nm, 350 μL m^−2^ s^−1^ intensity). The algal/CB-SPE was incubated for 10 min in the dark in a reaction volume of 200 mL of measuring buffer, and then stimulated by a light flash of 30 s of red LEDs, optically filtered to a peak wavelength of 650 nm. In this condition, the algal/CB-SPE generated peak current signals from 0.3 to 1.5 µA depending on the potential applied in the range from –0.8 to –0.3 V. As highlighted in Figure 5A, the ad hoc applied potential for the measurements light-induced oxygen evolution was equal to −0.6 V, which resulted in peak currents of 1.5 µA. This value was optimized using the best conditions obtained also for the light intensity and immobilized cell number, which were set to obtain the higher current signals at 350 μL photons m^−2^ s^−1^ intensity (Figure 5B) and 0.8 × 10^6^ immobilized cell number (Figure 5C), respectively.

### 3.3. Algal Cytosensor Analytical Response to Pathogens

**Figure 6:**
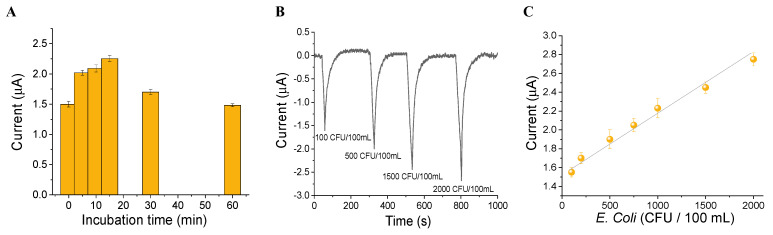
(**A**) Incubation time, (**B**) algal CB-SPEs amperogram after the addition of an increasing amount of *E. coli*, and (**C**) the corresponding calibration plot.

Algal/CB-SPEs were incubated in the dark with E. coli at a concentration of 1000 CFU/100 mL from 5 to 60 min, to evaluate the incubation time at which a higher algae oxygen production occurs due to the presence of bacteria, which reduce the photosynthetic oxygen tension within the microenvironment of the algal cells. Indeed, within a short incubation time from 5 and 15 min, an increase of the current signals was observed (Figure 6A), while at higher incubation time a balance of algal oxygen evolution and oxygen sequestration by bacteria was observed, thus providing current signals comparable to algae oxygen production in the absence of bacteria. Considering the results reported in Figure 6A, an incubation time of 15 min was selected.

To obtain a calibration curve for the detection of the target bacterium, algal/CB-SPEs were incubated for 15 min in the dark with E. coli in a concentration range from 100 to 2000 CFU/100 mL. Then, a light flash of 30 s of red LEDs (optically filtered to a peak wavelength of 650 nm at an intensity of 350 μL photons m^−2^ s^−1^) was applied to stimulate the algal photosynthetic light-induced oxygen evolution. An increase of the oxygen evolution and thus of the current signals was registered in the presence of the increasing pathogen concentration (Figure 6B), obtaining a linear response and allowing for the construction of a calibration curve described by the equation y = 1.530 (±0.059) − 0.00060 (±0.00005) x, with an R^2^ = 0.985 (Figure 6C). A detection limit of 92 CFU/100 mL was achieved (LOD = 3 × sd/slope). The linear range and the LOD found can be considered coherent with the maximum E. coli concentration suggested by Italian law for wastewater (less than 5000 CFU/100 mL) [25].

**Figure 7:**
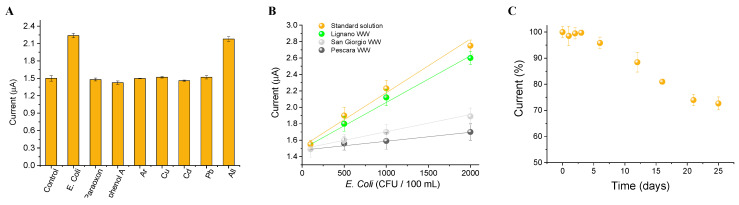
(**A**) Interference analysis using 10 ppb arsenic, 1.3 ppb copper, 5 ppb cadmium, 10 ppb lead, 10 ppb bisphenol A, and 1 ppb paraoxon, and a solution spiked with all the compounds. (**B**) Matrix effect using Lignano, San Giorgio, and Pescara wastewater samples. (**C**) Storage stability expressed as light-induced oxygen evolution.

To test the algal cytosensor in wastewater samples, its selectivity was evaluated analyzing metals (10 ppb arsenic, 1.3 ppb copper, 5 ppb cadmium, 10 ppb lead), pesticides (1 ppb paraoxon), and phenolic compounds (10 ppb bisphenol A) at legal limits established by the European legislations for surface water (where present) as interferents that should be present in wastewater from depuration plants [26]. The results reported in Figure 7A highlighted that the interfering species did not affect the analysis of E. coli at the tested concentrations, unless atrazine, which is, as a photosynthetic herbicide, the specific target of the alga. However, the presence of such a herbicide can also be analyzed by chlorophyll fluorescence simultaneously to the amperometric measurements, exploiting the optical module of the biosensor prototype (Figure 1), thus supporting the amperometric analysis of bacteria.

To investigate the suitability of the proposed cytosensor in real samples, the matrix effect was studied. Algal/CB-SPEs were incubated in 200 µL of 2X measuring buffer diluted 1:2 (*v:v*) in wastewater fortified with E. coli in a concentration range from 100 to 2000 CFU/100 mL. A calibration curve was obtained for each wastewater sample analyzed, described by the equations y = 1.49 (±0.05) − 0.0005 (±0.00004) × (R^2^ = 0. 989), y = 1.49 (±0.01) − 0.0001 (±0.00001) × (R^2^ = 0. 975), y = 1.48 (±0.01) − 0.0002 (±0.00001) × (R^2^ = 0.992) for Lignano, San Giorgio, and Pescara, respectively (Figure 7B). The ratio between the slopes of the calibration curves obtained in standard solutions and real samples was equal to 0.83, 0.16, 0.33, indicating a ~17, 84, and 67% dependence from Lignano, San Giorgio, and Pescara wastewater matrices, respectively. This is due to the presence of high concentration of ammoniacal nitrogen (N-NH_3_), nitrous nitrogen (N-NO_2_), nitric nitrogen (N-NO_3_), and dissolved inorganic nitrogen (DIN) found in the analyzed wastewater samples (i.e., 81.2 µg/L N-NH3, 831.1 µg/L N-NO_2_, 5212.3 µg/L N-NO_3_, and 6124.6 µg/L DIN in wastewater from Lignano depuration plant and 491.2 µg/L N-NH_3_, 56.1 µg/L N-NO_2_, 414.3 µg/L N-NO_3_, and 961.6 µg/L DIN in wastewater from San Giorgio depuration plant). The calibration curve obtained for the algal cytosensor in real samples was further used to calculate the recovery values of the surface water samples. Recovery values of 105 ± 8, 83 ± 7, and 88 ± 7% were obtained for 1000 CFU/100 mL of E. coli for Lignano, San Giorgio, and Pescara wastewater samples, respectively.

The storage stability of the algal cytosensor was evaluated by storing the algal/CB-SPEs in the measuring buffer at room temperature under continuous light at 50 μL photons m^−2^ s^−1^ for 21 days. Amperometric analysis at the defined time intervals of each SPE recorded in a 200 µL volume of measuring buffer showed that detectable loss of the photosynthetic activity occurred after 21 days (Figure 7C). Working stability was assessed by amperometric measurements run on the algal/CB-SPEs up to 12 h at room temperature, under repeated cycles of 10 min dark and 30 s light of red LEDs, showing 100% intra-electrode repeatability of light-induced oxygen evolution activity. Moreover, the preparation of the algal/CB-SPEs showed a high inter-electrode repeatability with RSD of 1.1% (n = 12) (data not shown).

## 4. Discussion and Conclusions

In this work, a cytosensor based on microalgae has been developed for the detection of *E. coli* presence in water outputs from wastewater treatment plants. The system, constituted of C. reinhardtii whole cells immobilized on the carbon black-modified working electrode of SPEs, detects the oxygen produced by the microalgae during the photosynthetic cycle when a potential of −0.6 V is applied in a chronoamperometry measurement. It has been demonstrated [24] that the presence of bacteria strains in growing microalgae solutions promote oxygen production, with the promotion effect not dependent by the specific bacteria strain. This phenomenon has been used in this case to develop a biosensor capable of quickly determining the presence of bacteria in water downstream from wastewater treatment plants. In particular, the assembled system can detect the presence of *E. coli* down to a LOD of 92 CFU/100 mL in real wastewater, with a linear response obtained in the range 100 to 2000 CFU/100 mL, after only 15 min of incubation time. The values found are in line with those obtained in similar works in the literature [27,28,29,30,31,32]. It should be underlined that this bacterium was exploited as a case study target of the algal cytosensor to demonstrate its ability as an early warning analytical system to signal heavy loads of pathogens in waters leaving the wastewater treatment plants. Indeed, the cytosensor is not selective towards E. coli but it is capable to sense all the bacteria that induce the algae oxygen evolution by exploiting the effect of their interaction. On the contrary, the cytosensor shows the advantages of not being affected from other pollutants commonly present in wastewaters and, in addition, any pretreatment of the sample is required before the analysis, resulting in an easy integration for on-line microorganism monitoring.

## Data Availability

Not applicable.
